# Protein expression profiles in hematopoietic stem/progenitor cells after exposure of mice to silicon (^28^Si) ions

**DOI:** 10.1093/jrr/rrt193

**Published:** 2014-03

**Authors:** Kanokporn Noy Rithidech, Montree Tungjai, Louise Honikel, Chris Gordon, Xianyin Lai, Frank Witzmann

**Affiliations:** 1 Department of Pathology, Stony Brook University, Stony Brook, NY 11794-8691, USA; 2 Department of Radiologic Technology, Faculty of Associated Medical Sciences, Chiang Mai University Chiang Mai, 50200 Thailand; 3 Department of Biochemistry and Molecular Biology, Indiana University, Indianapolis, IN 46202, USA; 4 Department of Cellular and Integrative Physiology, Indiana University, Indianapolis, IN 46202, USA

**Keywords:** protein expression profiles, hematopoietic stem cells, silicon ions

## Abstract

It has been well recognized that exposure to space radiation is a major challenge to space exploration. To protect astronauts in space environments, improvement in our knowledge of radiation-induced changes in specific target cells that may affect the health of astronauts is required. Cancer of blood cells, in particular myeloid leukemia (ML), is one of the major health concerns from exposure to space radiation. However, the predictions of risks for developing ML post-exposure to space radiation are unsatisfactory. To increase the reliability of predicting risk for ML, a much improved understanding of space radiation-induced changes in the target cells, i.e. hematopoietic stem/progenitor cells (HSPCs), is critically important. *In vivo* studies of humans are not possible. Thus, controlled and randomized animal experiments are critically important.

Most proteomic applications mentioned above have used 2-DE or stable isotope-tagged mass spectrometry. Although the 2-DE has several advantages (e.g. the detection of potential post-translational modifications of proteins which can be readily visualized on the gel, although the exact type of modification requires determination by mass spectrometry), such technology is simply not as comprehensive and reliable as desired. To overcome these limitations, we recently developed a unique label-free quantitative mass spectrometry (LFQMS) platform [ 1]. This is an innovative, experimentally based method that accurately determines peptide peak retention-time and uses multiple filters for exclusion of unqualified peptides by peptide frequency, retention time, intensity coefficient of variation and intensity correlation to enhance protein quantification of qualified peptides and proteins.

In this study, we used the LFQMS platform to examine protein expression-profiles in the colonies of HSPCs (the best population of cells for studying *in vivo* biological effects of radiation on hematopoietic stem cells) obtained at 6 months after exposure (at which radiation-induced genomic instability and chronic inflammation have been detected [ 2]) of CBA/CaJ mice whole-body to a total dose of 0, 0.1, 0.25 or 0.5 Gy of 300 MeV/nucleon ^28^Si ions, using a fractionated schedule (two exposures, 15 days apart that totaled each selected dose). These doses of 300 MeV/nucleon ^28^Si ions are comparable to what astronauts encounter in space. Mice exposed to 0 Gy of ^28^Si ions served as non-irradiated sham controls. The colonies of HSPCs were obtained from BM cells of five mice per treatment group, by means of an *in vitro* colony forming unit assay (CFU-A) using methylcellulose-based medium. Proteins were extracted from HSPC colonies and protein concentrations were determined by the Bradford Protein Assay. The trans-proteomic pipeline (TPP) was used to assign a probability of a particular protein being in the sample.

A total of 1344 unique, non-redundant proteins were identified with ≥90% confidence from 3254 peptides, quantified and their abundances were compared statistically. Among the 1344 proteins, differential expression of 198 proteins was found to be statistically significant in HSPC colonies obtained from treated groups, in relation to those found in non-irradiated sham controls. A proof-of-concept-based Ingenuity Pathway Analysis (IPA, www.ingenuity.com) was used to characterize the functions and pathways of these 198 identified proteins. The majority of these proteins are cancer-related (*P* < 0.0001). Biochemical analyses of the molecular and cellular functions of these proteins reveal association with perturbation in cell survival, free radical scavenging, cell cycle, DNA repair, cellular assembly, hematological system development and inflammatory responses. These proteins are linked to two major molecular networks that are linked to cancer and inflammatory responses (i.e. nuclear factor-κ B and the protein phosphatase 2 A networks). Our results indicated ^28^Si ion-induced damage in HSPCs.
